# Assessing the Effectiveness of Reproductive Health Literacy Trainings on Access To Care for Arab and Afghan Refugee Communities

**DOI:** 10.1007/s10903-025-01734-6

**Published:** 2025-07-16

**Authors:** Heike Thiel de Bocanegra, Asiya Yama, Ahmad Fahim Pirzada, Haidy Neamaallah, Jenny Chang, ReproNet Health Equity Workgroup

**Affiliations:** 1 Department of Obstetrics and Gynecology, School of Medicine, University of California, Irvine, Irvine, USA; 2 Department of Public Health Sciences, School of Medicine, University of California, Davis, Davis, USA; 3 Department of Public Health Sciences, School of Medicine, University of California, Davis, Davis, USA; 4 Department of Medicine, School of Medicine, University of California, Irvine, Irvine, USA

**Keywords:** Health literacy, Sexual and reproductive health, Refugees, Cervical cancer prevention, HLS-EU-Q6, eHEALS

## Abstract

This study assessed the impact of reproductive health literacy training for general, digital, and reproductive health literacy among Afghan and Arab refugees. From September 2023 to June 2024, the research team offered reproductive health literacy training (RHLT) series to Dari-, Pashto-, and Arabic-speaking refugees online and at public libraries in Sacramento, California. The RHLT series presented information on cervical cancer prevention, family planning, and maternal health with an emphasis on participants’ ability to independently find reliable health information and use it for their own and their families’ health decisions. A total of 203 participants completed the pre-assessment and post-assessment, administered two weeks after the final training session. Each assessment included validated scales, including the European Health Literacy Survey Questionnaire 6 (HLS-EU-Q6) and the eHealth Literacy Scale (eHEALS). Study participants in all three language groups significantly improved general health literacy, reproductive health literacy and reproductive knowledge (*p* < 0.05). Improvements in the digital health literacy scale (eHEALS) were borderline significant (*p* = 0.0506) and significant for Dari speakers (*p* < 0.05). There were no differences among the three language groups when controlling for age, education, and length of stay in the United States. These results showed that reproductive health literacy training that focuses on understanding and using health information is effective in increasing health literacy among Afghan and Arab refugee women with diverse cultural and demographic backgrounds. Scalable health literacy modules have the potential to reduce health disparities and empower informed decision-making within underserved populations.

**Supplementary Information:**

The online version contains supplementary material available at 10.1007/s10903-025-01734-6.

## Background

Between fiscal years 2014 and 2023, the United States resettled over 349,000 refugees^1^ and an additional 100,000 Afghans and Iraqis Special Immigrant Visa (SIV) holders. SIV holders faced serious threats due to their past employment with the U.S. government and/or U.S. military yet have the same benefits and support services as refugees [1]. Women and girls represent half of these displaced populations, often experiencing dire humanitarian conditions and heightened vulnerabilities which frequently result in unwanted pregnancies and sexually transmitted infections [2]. Refugee women face delays in accessing healthcare after resettling due to factors like socioeconomic challenges, cultural differences, language barriers, lack of knowledge about preventive care and the U.S. healthcare system, and stigma around seeking sexual health services beyond pregnancy-related issues [3, 4].

California is one of the top states for refugee arrivals in the U.S; from fiscal years 2012–2022, it accounted for approximately 9% of all refugee admissions [5]. Refugees came mainly from Arabic speaking countries such as Iraq, Syria, or Somalia and more recently from Afghanistan; they reflected a wide spectrum of cultural norms, socioeconomic backgrounds, and experiences [6–8]. Up to half of the recent refugee and SIV holder arrivals to California resettled in Sacramento county [9]. In the first wave of refugees to California, the majority of Afghan women spoke Dari, one of the two main commercial languages. However, since 2021, about two-thirds of refugee arrivals come from Pashto-speaking, conservative rural regions with lower literacy rates than other regions in Afghanistan [10–12].

The evaluation of health promotion interventions for refugees is often limited to qualitative assessments of feasibility and acceptability. These interventions lack data on refugees’ knowledge, health literacy, and behavior impact [13]. To address this gap, the Refugee Reproductive Health Network (ReproNet), based at the University of California, Irvine, developed a reproductive health literacy training (RHLT) curriculum to improve refugee women’s reproductive health knowledge and health literacy. Healthy People 2030’s definition of health literacy (“*ability to find*,* understand*,* and use information and services to inform health-related decisions and actions for themselves and others*”) [14] guided the development of the training. General health literacy and digital health literacy were found to be associated with health outcomes such as better ability to interpret health messages [15, 16], increased use of preventive services [17, 18]. and maternal health outcomes [19, 20].

This study describes the impact of eight RHLT series on refugee women’s general health literacy, digital health literacy, and reproductive health literacy.

## Methods

### Study Design

This study utilized a pre/post-assessment design to measure change in general, digital, and reproductive health literacy (outcome measures) and and compare the mean scores of women who spoke Arabic, Dari, and Pashto. Demographic data collected to describe the sample included age, country of birth, primary language, reading and writing abilities in both their primary language and in English, education level, type of health insurance, and number of children. Additionally, the research team evaluated changes in reproductive health knowledge from pre- to post-assessment. This study received approval from the University of California, Irvine Institutional Review Board #1443.

### Setting

ReproNet offered eight series of RHLTs between September 2023 and June 2024. Each series consisted of three two-hour weekly meetings, four at Sacramento Public Library (SPL) locations in neighborhoods with large Afghan and Arab populations, and four online via Zoom. Two series were offered in Pashto and one series each in Arabic and Dari. The facilitators, notetakers, and volunteers for these sessions were all female. The facilitators were multilingual, bicultural health professionals with experience in group facilitation who had received training on the RHLT objectives and didactic approach. They participated in a structured training that ReproNet staff led; it included an orientation on facilitation strategies, cultural sensitivity, and use of ReproNet’s reproductive health literacy training materials [21, 22]. Debriefing sessions were held to reinforce facilitator development and ensure consistency in delivery.

### RHLT Training

The RHLT sessions focused on topics such as cervical cancer prevention, including HPV vaccination, family planning, and contraception, as well as maternal health, pregnancy loss, and postpartum care. RHLT learning objectives included (a) Increasing knowledge of reproductive health; (b) Understanding the importance of cervical cancer and pregnancy preventive care; (c) Understanding the importance of self-care in the postpartum period; (d) Increasing comfort in communicating with health care professionals; and (d) Enhancing the ability to access and evaluate the accuracy of information sources (e.g., chat groups, social media). Participants were taught how to access the ReproNet digital library (www.repronet.org) [23], the interactive cervical cancer education tool [24], and video presentations [25–28]. They were encouraged to share these materials with their spouses or family members after the sessions and to explore additional digital resources on various reproductive health topics. Notetakers at each session completed evaluation forms to document content, questions and comments to monitor fidelity.

### Study Instrument

The study team developed a pre- and post-assessments that measured demographic information, reproductive health knowledge, and three health literacy domains: general health literacy, digital health literacy, and reproductive health literacy [29]. For changes in reproductive health knowledge, we created three to four questions per session topic (cervical cancer prevention, family planning, and maternal health). We identified validated scales with a low literacy level that were used in different languages and refugee and native populations (Table 1) and assessed validity and reliability with our study population [29]. The European Health Literacy Survey Questionnaire 6 (HLS-EU-Q6) [30, 31] measured GHL and the eHealth Literacy Scale (e-HEALS) [32] assessed DHL. The RHL scale is a composite of selected items from the Cervical Cancer Literacy Assessment Tool (C-CLAT) [33], the UCI Postpartum Health Literacy Scale [34], and the Iranian Adult Sexual Health Literacy Assessment Standard Questionnaire (SHELA) [35].

**Table 1:** Reproductive health literacy scale items. Summarizes the reproductive health literacy scale items across three domains: general health literacy (HLS-EU-Q6), digital health literacy (e-HEALS), and reproductive health literacy (C-CLAT and SHELA), highlighting their validation, item counts, and alpha coefficients

	Domain		Scale Name	Number of items, where used	
	General Health Literacy		European Health Literacy Survey Questionnaire 6 (HLS-EU-Q6)[30,31]	Shorter version of the 47-item (HLS-EU-Q47) and 16-item (HLS-EU-Q16). Widely used and validated in multiple languages, including Dari, Pashto, and Arabic. The HLS-EU-Q6 is highly correlated with the HLS-EU-Q47 (*r* = 0.896) and has an alpha coefficient of 0.803.	
	Digital Health Literacy		e-Health Literacy Scale (e-HEALS)[32]	Measures abilities to locate, understand, and apply electronic health information. 8-item scale, alpha coefficient of 0.88 and factor loadings between 0.60 and 0.8421. The scale has been translated into Arabic and validated for use with Arabic-speaking populations, including Syrian and Iraqi migrants (α = 0.92).	
	Reproductive Health Literacy				
		Cervical Cancer	Cervical Cancer Literacy Assessment Tool (C-CLAT)[33]	Has strong factor loadings and was validated in Arabic. Two C-CLAT questions (Q1, Q10) were modified to emphasize understanding and applying cervical cancer knowledge (α = 0.89).	
		Family Planning & Sexual Health	Iranian Adult Sexual Health Literacy Assessment Standard Questionnaire (SHELA)[35]	Was validated in Farsi/Dari with alpha coefficient of 0.88. Adapted three items: Q5 (“I can obtain information on various methods of pregnancy prevention”), Q30 (“I can identify where to seek help for sexual problems or disorders)	
Postpartum			UCI Postpartum health literacy scale [34]	Selected five items (Q 17, Q20, Q21, Q22, Q24) from a postpartum health literacy scale developed and tested with Arabic immigrants and SHELA Q23 (“I know when postpartum symptoms require medical attention”)	

The study team translated items that were not available in Dari, Pashto and/or Arabic. Native speakers with medical interpretation training reviewed and tested the final translations for errors, quality, and cultural appropriateness. The translations were also piloted among women in each language group to ensure clarity and understanding. The scales had Cronbach alpha coefficients between 0.82 and 0.93, indicating acceptable internal consistency. Details on the scale development and testing are reported in elsewhere [29]. The assessments took on average 10–15 min to complete; in addition, the pre-assessment had demographic questions and creation of a participant code.

### Participant Recruitment and Study Outreach

Participants were recruited through bilingual flyers with registration links and QR codes, distributed at public libraries, community events, on social media and through targeted outreach in collaboration with refugee networks in Sacramento County. Recruitment was open to anyone who used these services, regardless of place of residence. The most effective strategy was direct telephone calls and text messages from staff of the Muslim American Society – Social Services Foundation (MAS-SSF) to their client and event contact list. The inclusion criteria were Dari, Pashto and Arab-speaking refugee/ immigrant women aged 18 years or older.

### Pre- and Post-Assessment Administration

At SPL library sessions, participants completed the pre-assessment using paper forms; they also received a hard copy of the consent form at their first session. Walk-in participants could register on-site. Childcare services for preschool-aged children and refreshments were provided, allowing for informal interactions after the sessions.

For the online sessions, participants pre-registered and received links to the Zoom meeting and the REDCap (Research Electronic Data Capture) pre-assessment [36] via email. They also received the consent form in their primary language as an attachment. Participants completed the pre-assessment before the first session. During the sessions, coordinators confirmed that all participants were female. Sessions had tech support to assist with log-in and navigating the platform. Some participants shared devices within the same household.

In both formats, trained bilingual staff provided assistance to complete the pre- and post-assessments, if needed. Post-assessments were administered two weeks after the final session, and participants were reminded up to three times to complete them; this resulted in a two-month gap between pre- and post-assessment. In-person participants had the option to complete the survey via REDCap or in-person at the MAS-SSF office.

Participants received stipends for attendance and completion of the pre- and post-assessments. For their time, in-person training participants received $20 when they completed the pre-assessment and at each of the three sessions and online participants $30 gift card if they completed pre- and post-assessments.

### Statistical Analysis

For the full sample and each language group, summary statistics, including the mean and standard deviation, were calculated for each question and scale at pre-test, post-test, and for change. Since the study used a pre/post design, a paired t-test was used to account for individual differences. By comparing scores from the same participants before and after the intervention, this approach reduced the impact of baseline variability. Additionally, multivariate linear regression was conducted to examine the association between the outcome (change in score) and participants’ demographic characteristics.

The final analytic sample included only participants who completed both the pre- and post-intervention questionnaires. Because the analysis relied on paired t-tests and the outcome for multivariable analysis was change in score, missing data were not a major concern. All statistical analyses were performed using SAS 9.4 [37]. Statistical significance was set at *P* < 0.05, using two-tailed tests.

## Results

Of the 237 participants who completed the pre-test, 203 (85.7%) completed the post-test and were included in the primary analysis. In the pre-assessments, there were no significant differences between completers (*n* = 203) and non-completers (*n* = 34) by demographics. Retention rates by language group were high but varied by language group with Pashto-speaking participants at 80%, Dari-speakers at 89%, and Arabic-speakers at 94%. (*p* = 0.0321) (Supplementary Table 1).

The mean age of the participants was 35.1 years, with Arabic speakers averaging 36.3 years, making them the oldest group; Pashto speakers averaged 34.6 years of age. On average, the participants had been in the U.S. for 4.6 years. The duration of residence in the U.S. was notably significant (*p* = 0.0063), with Arabic speakers residing the longest (6.7 years), followed by Dari speakers (4.1 years) and Pashto speakers (3.9 years). Nearly two-thirds of the cohort (63.5%) were married or in domestic partnerships, with the highest proportion among Dari speakers (81%). The average number of children was 3.1 with Pashto speakers having the highest average number of children (3.4). (Table 2)

**Table 2:** Demographic characteristics of training participants who completed both pre- and post-tests (*n* = 203) including age distribution, marital status, and level of education; the table shows that there was no statistically significant differences in age, marital status, education level, english or preferred Language literacy, insurance status, or country of birth. However, significant differences emerged by race/ethnicity (*p* = 0.0262) and preferred Language (*p* = 0.0321)

	Total (n = 203)		Pashto (n = 102)		Dari (n = 51)		Arabic (n = 50)		*p*-value*
	N	%	N	Col %	N	Col %	N	Col %	
Age at Pretest (years)									0.0432
18–29	70	34.5	34	33.3	17	33.3	19	38	
30–39	67	33	40	39.2	19	37.3	8	16	
40+	66	32.5	28	27.5	15	29.4	23	46	
Marital status									0.1388
Single/divorced/widowed	44	21.7	20	23	8	19	16	36.4	
Married	129	63.5	67	77	34	81	28	63.6	
Number of children									0.2833
0	44	21.7	20	20	10	19.6	14	28	
1–2	39	19.2	21	21	12	23.5	6	12	
3–4	59	29.1	24	24	16	31.4	19	38	
5+	59	29.1	35	35	13	25.5	11	22	
Insurance									0.0018
Public	178	87.7	97	95.1	44	86.3	37	74	
Private	12	5.9	2	2	2	3.9	8	16	
Other/unknown	13	6.4	3	2.9	5	9.8	5	10	
Education									< 0.0001
Never attended school – 6th grade	51	26.3	37	39.4	11	22	3	6	
7–12 years of schooling	103	53.1	48	51.1	30	60	25	50	
College or above	40	20.6	9	9.6	9	18	22	44	
Able to read and write English									0.0082
Both read and write	102	51.3	51	52	22	43.1	29	58	
Either read or write	34	17.1	9	9.2	12	23.5	13	26	
Neither	63	31.7	38	38.8	17	33.3	8	16	
Able to read and write in preferred language									0.0003
Both read and write	145	72.5	61	61	37	74	47	94	
Either read or write	24	12	14	14	8	16	2	4	
Neither	31	15.5	25	25	5	10	1	2	
In US > 5 years									0.0063
No	87	42.9	53	52	22	43.1	12	24	
Yes	42	20.7	15	14.7	9	17.6	18	36	
Unknown	74	36.5	34	33.3	20	39.2	20	40	
Country of birth (n = 178)									< 0.0001
Afghanistan	131	73.6	85	96.6	46	95.8	0	0	
Syria	32	18	0	0	0	0	32	76.2	
United States	4	2	0	0	0	0	4	9.5	
Other**	11	5.4	3	3	2	4	6	12	
Race/ethnicity									< 0.0001
Asian	144	70.9	89	87.2	47	92.2	8	16	
Middle Eastern/North African (MENA)	35	17.2	0	0	1	2	35	70	
Non-Hispanic White	16	7.9	9	8.8	1	2	6	12	
Non-Hispanic Black	1	0.5	1	1	0	0	0	0	
Prefer not to respond	7	3.4	4	3.9	2	3.9	1	2	

*p-value from Chi square test or Fisher’s exact test or two sample t test for difference among groups

**Other—Iraq, Egypt, Palestine, Algeria, UAE, Iran, Russia, Pakistan

Insurance coverage also varied significantly among the three groups (*p* = 0.0001): the majority of participants (86%) had Medi-Cal health insurance (California’s Medicaid program). Only Arabic- and Dari-speaking participants had private insurance coverage (21% and 11%, respectively); a small number of participants (6%) reported having no insurance. (Table 2)

Three-fourths (76.2%) of the Arabic speakers were born in Syria. Among the 50 Arabic-speaking participants, 30 participants (60%) identified as Middle Eastern/North African (MENA). The large majority of the Dari and Pashto-speakers were from Afghanistan and identified as Asian. The rest of the study cohort self-identified as White, MENA, or Black/other race. (Table 2)

Self-reported English proficiency varied significantly (*p* = 0.0082), with 50.2% of participants reporting ability to read and write in English; the percentage of respondents ranged from 58% Arabic speakers to 39% Pashto speakers. Reading and writing ability in the preferred language also varied significantly (*p* = 0.0001), with Arabic speakers reporting the highest literacy rate (94%) compared with 74% for Dari speakers and 61% for Pashto speakers. Similarly, education levels were significantly higher among Arabic speakers (*p* < 0.0001), with 44% having attended some college or higher, contrasted with just 10% of Pashto speakers. (Table 2)

### Changes in Pre- /Post-Assessment Scores

Pre- and post-assessment comparisons demonstrated statistically significant improvements across all primary outcome domains (Table 3). In the total sample, all items improved from pre- to post-assessment. The health literacy score improved from a mean of 2.64 (SD 0.7) at pre-assessment to 2.95 (SD 0.6) at post-assessment (*p* < 0.0001); the reproductive health literacy score increased from 2.63 to 2.91 (*p* < 0.0001); and reproductive health knowledge score rose from 0.35 to 0.55 (*p* < 0.0001). A smaller, borderline-significant improvement was observed in the digital health literacy score (2.67 to 2.77; *p* = 0.0506).

**Table 3:** Mean and standard deviations of health literacy and knowledge scales, and paired t-test results: total sample and by Language group

Group	Scale	Pre-Test Mean (SD)	Post-Test Mean (SD)	Change (Post–Pre) (SD)	Paired t-test *p*-value
**Total (** ***n*** ** = 203)**	Health Literacy	2.64 (0.7)	2.95 (0.6)	0.31 (0.7)	< 0.0001
	Digital Health Literacy	2.67 (0.7)	2.77 (0.6)	0.10 (0.7)	0.0506
	Reproductive HL	2.63 (0.8)	2.91 (0.6)	0.28 (0.8)	< 0.0001
	Reproductive Knowledge	0.35 (0.3)	0.55 (0.3)	0.20 (0.3)	< 0.0001
**Pashto (** ***n*** ** = 102)**	Health Literacy	2.77 (0.7)	3.04 (0.6)	0.28 (0.8)	0.0008
	Digital Health Literacy	2.70 (0.7)	2.75 (0.7)	0.04 (0.8)	0.6392
	Reproductive HL	2.62 (0.8)	2.82 (0.6)	0.20 (0.9)	0.0235
	Reproductive Knowledge	0.30 (0.3)	0.48 (0.3)	0.19 (0.3)	< 0.0001
**Dari (** ***n*** ** = 51)**	Health Literacy	2.45 (0.7)	2.88 (0.7)	0.44 (0.8)	0.0002
	Digital Health Literacy	2.66 (0.7)	2.91 (0.6)	0.25 (0.7)	0.0191
	Reproductive HL	2.71 (0.9)	3.00 (0.6)	0.32 (1.0)	0.0334
	Reproductive Knowledge	0.28 (0.3)	0.56 (0.3)	0.27 (0.3)	< 0.0001
**Arabic (** ***n*** ** = 50)**	Health Literacy	2.58 (0.5)	2.82 (0.4)	0.25 (0.4)	< 0.0001
	Digital Health Literacy	2.62 (0.5)	2.70 (0.5)	0.08 (0.5)	0.2799
	Reproductive HL	2.58 (0.5)	2.98 (0.4)	0.40 (0.6)	< 0.0001
	Reproductive Knowledge	0.52 (0.3)	0.67 (0.3)	0.16 (0.3)	0.0009

When analyzed by language group, all three groups demonstrated gains in health literacy and reproductive health knowledge, though the magnitude of change varied. Dari speakers showed the greatest increase in general health literacy (+ 0.44, *p* = 0.0002), reproductive health knowledge (+ 0.27, *p* < 0.0001), and digital health literacy (+ 0.25, *p* = 0.0191) compared to the Arab and Pashto speakers (Table 3).

In the adjusted models, the study team did not find any differences between language groups when controlling for age, education, and length of stay in the U.S (Table 4).

**Table 4:** Multivariate regression model for Pre–Post test change of scales by Language. *Model adjusted for age, education, and length of stay in the U.S. (under 5 years vs. 5 years or more)

Outcome	Language	Adjusted Estimate	S.E.	*P*-Value
**Health Literacy**	Pashto	Ref	–	–
	Dari	0.19	0.13	0.1321
	Arabic	-0.05	0.14	0.7442
**Digital Health Literacy**	Pashto	Ref	**–**	**–**
	Dari	0.18	0.13	0.1598
	Arabic	0.04	0.14	0.7852
**Reproductive HL**	Pashto	Ref	–	–
	Dari	0.18	0.15	0.2307
	Arabic	0.22	0.16	0.1638
**Reproductive Knowledge**	Pashto	Ref	–	–
	Dari	0.10	0.06	0.0976
	Arabic	0.02	0.06	0.7785

## Discussion

Initiatives to improve health literacy aim to empower individuals to make informed decisions about their own health and the well-being of their families [14]. The term “e-health literacy” was first coined in 2006 [38], and intervention studies have demonstrated the effectiveness of health literacy and e-health or digital health literacy interventions for the management of chronic diseases [39, 40]. However, studies reporting “reproductive health literacy” improvements measured only improvements of reproductive health knowledge after sexual and reproductive health topics were integrated into literacy classes [41]. We found only one focus group study on family planning literacy that aimed to improve participants’ ability to seek, understand, and use health information [42, 43].

The main goal of this study is to apply the Healthy People 2030 definition of health literacy— “*the ability to find*,* understand*,* and use information and services to inform health-related decisions and actions for themselves and others*” [14] —to reproductive health issues in refugee women and evaluate its effectiveness through pre- and post-assessments. Observations and group feedback after the sessions revealed that participants became more comfortable discussing sexual and reproductive health topics, which are often considered taboo or private. Increased normalization of these topics and more open discussions were evident as participants actively engaged in the sessions and encouraged female friends and family to attend subsequent sessions or additional RHLT series. Due to the widespread use of virtual meetings during and after the COVID-19 pandemic, participants were familiar and comfortable with virtual meetings. We could equally engage participants in in-person and in online RHLT sessions. However, it was important that the sessions were limited to female attendees, including female facilitators and notetakers.

Reproductive health literacy training (RHLT), focusing on identifying reliable health information sources, prevention, communication with clinicians, and self-care, significantly improved Arab and Afghan women’s reproductive health knowledge, general health literacy, and reproductive health literacy. Language did not affect the RHLT’s effectiveness when accounting for education level, age, and length of stay in the U.S., indicating its suitability for refugee women from diverse backgrounds. Digital health literacy improved for all groups; however, the change was only statistically significant for the Dari-speaking Afghan refugees. Our observation that there is variability among individuals by country/region of origin and that individuals with lower education and older age have lower health literacy scores is consistent with assessments of the original HLS-EU-Q47 [44]. However, the impact of the reproductive health literacy training on health care seeking behaviors and health outcomes will need to be addressed.

Although the pre- and post-assessments were essentially the same, it is unlikely that participants remembered their previous responses after two months. As all assessments were conducted in participants’ native languages, score improvements are unlikely due to any increased English proficiency. Instead, the study findings —along with participants’ verbal confirmation of increased knowledge and assertiveness—suggest that gains were due to meaningful improvements in understanding specific reproductive health topics and reproductive health literacy overall.

Cultural norms may dictate how individuals seek information (e.g., through elders, religious leaders, family, female friends) versus seeking the information on their own via the internet [45]. The RHLT encouraged participants to watch the ReproNet videos and other resources on the ReproNet digital library with their partner, family members, and friends. Involving others may also support health literacy skills among participants with limited reading and writing skills who may require more time and practice and use digital health resources [46]. 

Our study aimed to enhance health awareness and literacy among vulnerable refugee women who face significant barriers to care and often have worse health outcomes than women in the general U.S. population. This aligns with the Healthy People 2030 goal of eliminating health disparities, achieving health equity, and improving health literacy to enhance the well-being of all individuals [47]. The RHLT is also consistent with the National Library of Medicine’s strategic plan to engage a wide range of audiences to ensure that information gets delivered to them at the right time [48]. ReproNet implements a digital library with curated educational materials on reproductive health in different languages [repronet.org] and resources for facilitators that can be used by a wide group of people diverse in age, educational level, and ethnic backgrounds and in a variety of settings such as refugee resettlement and community programs, primary care clinics, and English as Second Language-classes.

### Limitations

This study has several limitations. The in-person survey completion support for emerging readers or participants unfamiliar with survey administration risks the introduction of a response bias. We addressed this through rigorous training of research staff. Despite the use of validated scales and thorough quality check of translations, the meaning of some items may have been unfamiliar to the respondents. For example, research staff commented that some participants responded to the question “would you consult a second opinion?” (HLS-EU-Q6. Scale item 1) with the comment “I would consult my husband.” Due to limited resources, we were not able to observe the impact of the RHLT on the health care seeking behaviors and decisions. However, health literacy studies suggest that improved health literacy will lead to improved preventive health behavior and informed decision-making on treatment options [49, 50].

## Conclusion

Our study demonstrated that refugee women are eager to discuss sexual and reproductive health topics in in-person and online sessions when delivered by content experts in a trusted environment. RHLT is a promising tool to engage refugee women from different demographic and cultural backgrounds in clinical encounters, health decisions, preventive health behaviors, and self-care. Reproductive health promotion and prevention programs should be designed within a comprehensive health literacy framework to achieve optimal effectiveness and health equity [44, 51].

## Electronic Supplementary Material

Below is the link to the electronic supplementary material.

Supplementary Material 1

Supplementary Material 2

Supplementary Material 3

## Data Availability

The data that support the findings of this study are not openly available due to reasons of sensitivity and are available from the corresponding author upon reasonable request.

## Abbreviations

RHLT: Reproductive Health Literacy Training
SIV: Special Immigrant Visa
ReproNet: Refugee Reproductive Health Network
MAS-SSF: Muslim American Society-Social Services Foundation
SPL: Sacramento Public Library
HLS-EU-Q6: European Health Literacy Survey Questionnaire 6
HLS-EU-Q47: European Health Literacy Survey Questionnaire 47
HLS-EU-Q16: European Health Literacy Survey Questionnaire 16
eHEALS: e-Health Literacy Scale
C-CLAT: Cervical Cancer Literacy Assessment Tool
SHELA: Iranian Adult Sexual Health Literacy Assessment Standard Questionnaire

## Footnotes

1 In this paper, “refugee” refers to refugees, SIV holders, parolees, and asylees.
