# Supplementary material for: Assessing the Effectiveness of Reproductive Health Literacy Trainings on Access To Care for Arab and Afghan Refugee Communities
Source: J Immigr Minor Health. 2025 Jul 16;27(6):967–76. doi: 10.1007/s10903-025-01734-6 (PMC12599836; doi:10.1007/s10903-025-01734-6)
Supplement: Supplementary file 1 — Supplementary Material 1 [file 10903_2025_1734_MOESM1_ESM.docx]

| **Supplementary Table 1. Comparison of pretests of those who completed did not complete pretest with those who did** | | Total (n=237) | | No follow up (n=34) | | Had follow up (n=203) | | p-value* |
| --- | --- | --- | --- | --- | --- | --- | --- | --- |
|  |  | N | % | N | Row % | N | Row % |  |
| Age at Pretest (years) | |  |  |  |  |  |  | 0.1038 |
| 18-29 | | 81 | 34.2% | 11 | 13.6% | 70 | 86.4% |  |
| 30-39 | | 84 | 35.4% | 17 | 20.2% | 67 | 79.8% |  |
| 40+ | | 72 | 30.4% | 6 | 8.3% | 66 | 91.7% |  |
| Marital status | |  |  |  |  |  |  | 0.8066 |
| Single/divorced/widowed | | 51 | 21.5% | 7 | 13.7% | 44 | 86.3% |  |
| Married | | 152 | 64.1% | 23 | 15.1% | 129 | 84.9% |  |
| Able to read and write English | |  |  |  |  |  |  | 0.7600 |
| Only read | | 29 | 12.2% | 6 | 20.7% | 23 | 79.3% |  |
| Only write | | 13 | 5.5% | 2 | 15.4% | 11 | 84.6% |  |
| Both read and write | | 117 | 49.4% | 15 | 12.8% | 102 | 87.2% |  |
| Neither | | 74 | 31.2% | 11 | 14.9% | 63 | 85.1% |  |
| Able to read and write in preferred language | |  |  |  |  |  |  | 0.4640 |
|  | Only read | 17 | 7.2% | 2 | 11.8% | 15 | 88.2% |  |
|  | Only write | 10 | 4.2% | 1 | 10.0% | 9 | 90.0% |  |
|  | Both read and write | 167 | 70.5% | 22 | 13.2% | 145 | 86.8% |  |
|  | Neither | 40 | 16.9% | 9 | 22.5% | 31 | 77.5% |  |
| Education | |  |  |  |  |  |  | 0.1526 |
|  | Up to high school | 183 | 77.2% | 29 | 15.8% | 154 | 84.2% |  |
|  | Some college or above | 43 | 18.1% | 3 | 7.0% | 40 | 93.0% |  |
| In US >5 years | |  |  |  |  |  |  | 0.2233 |
|  | No | 107 | 45.1% | 20 | 18.7% | 87 | 81.3% |  |
|  | Yes | 47 | 19.8% | 5 | 10.6% | 42 | 89.4% |  |
|  | Unknown | 83 | 35.0% | 9 | 10.8% | 74 | 89.2% |  |
| Number of children | |  |  |  |  |  |  | 0.2293 |
|  | 0 | 53 | 22.4% | 9 | 17.0% | 44 | 83.0% |  |
|  | 1-2 | 41 | 17.3% | 2 | 4.9% | 39 | 95.1% |  |
|  | 3-4 | 71 | 30.0% | 12 | 16.9% | 59 | 83.1% |  |
|  | 5+ | 69 | 29.1% | 10 | 14.5% | 59 | 85.5% |  |
| Race/ethnicity | |  |  |  |  |  |  | 0.0262 |
|  | MENA | 33 | 13.9% | 1 | 3.0% | 32 | 97.0% |  |
|  | Non-Hispanic White Caucasian | 16 | 6.8% | 0 | 0.0% | 16 | 100.0% |  |
|  | Non-Hispanic Black | 2 | 0.8% | 1 | 50.0% | 1 | 50.0% |  |
|  | Hispanic | 1 | 0.4% | 0 | 0.0% | 1 | 100.0% |  |
|  | Asian | 161 | 67.9% | 26 | 16.1% | 135 | 83.9% |  |
|  | Others | 24 | 10.1% | 6 | 25.0% | 18 | 75.0% |  |
| Insurance | |  |  |  |  |  |  | 0.9499 |
|  | Medicaid/MediCal | 204 | 86.1% | 30 | 14.7% | 174 | 85.3% |  |
|  | Emergency or pregnancy-related Medicaid | 2 | 0.8% | 0 | 0.0% | 2 | 100.0% |  |
|  | Private insurance | 12 | 5.1% | 1 | 8.3% | 11 | 91.7% |  |
|  | Not applicable/No health insurance | 7 | 3.0% | 1 | 14.3% | 6 | 85.7% |  |
|  | Other | 7 | 3.0% | 1 | 14.3% | 6 | 85.7% |  |
| Country of birth | |  |  |  |  |  |  | 0.2600 |
| United States | | 5 | 2.1% | 1 | 20.0% | 4 | 80.0% |  |
| Afghanistan | | 160 | 67.5% | 29 | 18.1% | 131 | 81.9% |  |
| Syria | | 33 | 13.9% | 1 | 3.0% | 32 | 97.0% |  |
| Other | | 11 | 4.6% | 0 | 0.0% | 11 | 100.0% |  |
| Preferred language | |  |  |  |  |  |  | 0.0321 |
|  | Pashto | 127 | 53.6% | 25 | 19.7% | 102 | 80.3% |  |
|  | Dari | 57 | 24.1% | 6 | 10.5% | 51 | 89.5% |  |
|  | Arabic | 53 | 22.4% | 3 | 5.7% | 50 | 94.3% |  |
| Setting | |  |  |  |  |  |  | 0.5978 |
|  | In Person | 88 | 37.1% | 14 | 15.9% | 74 | 84.1% |  |
|  | Online | 149 | 62.9% | 20 | 13.4% | 129 | 86.6% |  |
|  |  | Mean | SD | Mean | SD | Mean | SD |  |
| Age at Pre test (years) | | 34.7 | 10.7 | 31.9 | 8.1 | 35.1 | 11.0 | 0.0970 |
| Number of children | | 3.2 | 2.5 | 3.3 | 2.6 | 3.1 | 2.4 | 0.7410 |
| Age at immigration (years) | | 31.0 | 12.5 | 29.4 | 9.3 | 31.3 | 13.1 | 0.4864 |
| Length of time in US (years) | | 4.5 | 6.7 | 4.2 | 4.8 | 4.6 | 7.0 | 0.7913 |
| HLS-EU-Q6​ | | 2.6 | 0.7 | 2.5 | 0.7 | 2.6 | 0.7 | 0.3049 |
| eHeals​ | | 2.7 | 0.7 | 2.6 | 0.8 | 2.7 | 0.7 | 0.5245 |
| RHL | | 2.6 | 0.8 | 2.3 | 0.9 | 2.6 | 0.8 | 0.0320 |
| Knowledge | | 0.4 | 0.3 | 0.4 | 0.3 | 0.3 | 0.3 | 0.3888 |
| *p-value from Chi square test or Fisher's exact test or two sample t test for difference between follow up status | | | | | | | | |
