# Supplementary material for: Assessing the Effectiveness of Reproductive Health Literacy Trainings on Access To Care for Arab and Afghan Refugee Communities
Source: J Immigr Minor Health. 2025 Jul 16;27(6):967–76. doi: 10.1007/s10903-025-01734-6 (PMC12599836; doi:10.1007/s10903-025-01734-6)
Supplement: Supplementary file 2 — Supplementary Material 2 [file 10903_2025_1734_MOESM2_ESM.docx]

| **Supplementary Table 2: Detailed model between pre- and post-test scores when controlling for co-variates.** | **Change of Average of General Health Literacy** | | | **Change of average of digital health literacy** | | |
| --- | --- | --- | --- | --- | --- | --- |
|  | Adjusted Estimate | S.E. | P-Value | Adjusted Estimate | S.E. | P-Value |
| **Age (Years)** | -0.0039 | 0.0047 | 0.4096 | 0.0001 | 0.0048 | 0.9825 |
| **Preferred Language** |  | | |  | | |
| Pashto | Ref | | | Ref | | |
| Dari | 0.19 | 0.13 | 0.1321 | 0.18 | 0.13 | 0.1598 |
| Arabic | -0.05 | 0.14 | 0.7442 | 0.04 | 0.14 | 0.7852 |
| **In US >5 years** |  | | |  | | |
| No | Ref | | | Ref | | |
| Yes | 0.03 | 0.14 | 0.8181 | -0.10 | 0.15 | 0.5120 |
| Unknown | -0.06 | 0.12 | 0.6273 | 0.13 | 0.12 | 0.2989 |
| **Education** |  | | |  | | |
| Up to High School | Ref | | | Ref | | |
| Some college or above | 0.06 | 0.14 | 0.6628 | 0.06 | 0.14 | 0.6449 |
