# Supplementary material for: Assessing the Effectiveness of Reproductive Health Literacy Trainings on Access To Care for Arab and Afghan Refugee Communities
Source: J Immigr Minor Health. 2025 Jul 16;27(6):967–76. doi: 10.1007/s10903-025-01734-6 (PMC12599836; doi:10.1007/s10903-025-01734-6)
Supplement: Supplementary file 3 — Supplementary Material 3 [file 10903_2025_1734_MOESM3_ESM.docx]

**Pre- and Post-Intervention Scores on Reproductive Health Literacy Scale Items (HLS-EU-Q6, eHEALS, and Reproductive Health Literacy)**

| **HLS-EU-Q6:** On a scale from very easy to very difficult, how easy would you say it is to… | | **Total Pre Mean** | **Total SD** | **Total Post**  **Mean** | **Total**  **Post**  **SD** | **Change Mean** |
| --- | --- | --- | --- | --- | --- | --- |
| 1 | judge when you may need to get a second opinion from another doctor. | 2.55 | 0.90 | 2.83 | 0.82 | 0.30 |
| 2 | use information the doctor gives you to make decisions about your illness. | 2.71 | 0.84 | 3.04 | 0.73 | 0.35 |
| 3 | find information on how to manage mental health problems like stress or depression. | 2.50 | 0.89 | 2.92 | 0.83 | 0.42 |
| 4 | judge if the information on health risks in the media is reliable. | 2.57 | 0.89 | 2.79 | 0.83 | 0.22 |
| 5 | find out about activities that are good for your mental well-being. | 2.71 | 0.95 | 3.01 | 0.78 | 0.31 |
| 6 | understand information in the media on how to get healthier. | 2.82 | 0.83 | 3.10 | 0.74 | 0.28 |
| **eHEALS** | | **Total**  **Pre Mean** | **Total**  **Pre**  **SD** | **Total Post**  **Mean** | **Total Post SD** | **Change**  **Mean** |
| 7 | I know how to find helpful health resources on the Internet. | 2.78 | 0.86 | 2.86 | 0.71 | 0.08 |
| 8 | I feel confident in using information from the Internet to make health decisions. | 2.69 | 0.82 | 2.78 | 0.75 | 0.10 |
| 9 | I know where to find helpful health resources on the Internet. | 2.65 | 0.83 | 2.82 | 0.76 | 0.17 |
| 10 | I know how to use the Internet to answer my health questions. | 2.72 | 0.83 | 2.80 | 0.75 | 0.07 |
| 11 | I know what health resources are available on the Internet. | 2.68 | 0.81 | 2.79 | 0.77 | 0.11 |
| 12 | I can tell high quality from low quality health resources on the Internet. | 2.56 | 0.80 | 2.70 | 0.74 | 0.13 |
| 13 | I know how to use the health information I find on the Internet to help me. | 2.70 | 0.81 | 2.80 | 0.74 | 0.10 |
| 14 | I have the skills I need to evaluate the health resources I find on the Internet. | 2.65 | 0.80 | 2.72 | 0.73 | 0.05 |
| **Reproductive Health Literacy** | | **Total**  **Pre Mean** | **Total**  **Pre**  **SD** | **Total**  **Post**  **Mean** | **Total Post**  **SD** | **Change Mean** |
| 15 | I know what I can do to prevent cervical cancer. | 2.33 | 1.03 | 2.72 | 0.78 | 0.40 |
| 16 | I understand what can be done if I have an abnormal cervical cancer test. | 2.40 | 1.00 | 2.74 | 0.78 | 0.33 |
| 17 | I understand how the reproductive system works. | 2.48 | 1.02 | 2.81 | 0.73 | 0.34 |
| 18 | As soon as I realize a sexual problem or disorder, I can find out where or to whom I should go. | 2.68 | 1.00 | 2.94 | 0.68 | 0.26 |
| 19 | I can obtain information on various methods of pregnancy prevention from various sources. | 2.76 | 0.97 | 3.03 | 0.66 | 0.28 |
| 20 | I know how to get information about healthy nutrition before getting pregnant. | 2.72 | 1.00 | 3.03 | 0.64 | 0.31 |
| 21 | I know when and where to go for the necessary tests or examinations when I am pregnant. | 2.94 | 0.91 | 3.01 | 0.67 | 0.08 |
| 22 | I can find out how to schedule check-ups for myself or a friend after giving birth. | 2.65 | 1.00 | 2.92 | 0.69 | 0.28 |
| 23 | I know when symptoms after giving birth are so severe that I should see a doctor. | 2.78 | 0.96 | 3.05 | 0.64 | 0.28 |
| 24 | I know how to identify symptoms of postpartum depression. | 2.67 | 1.01 | 2.91 | 0.72 | 0.24 |
